# Monensin Alters the Functional and Metabolomic Profile of Rumen Microbiota in Beef Cattle

**DOI:** 10.3390/ani8110211

**Published:** 2018-11-17

**Authors:** Ibukun Ogunade, Hank Schweickart, Kenneth Andries, Jerusha Lay, James Adeyemi

**Affiliations:** College of Agriculture, Communities, and the Environment, Kentucky State University, Frankfort, KY 40601, USA; hank.schweickart@kysu.edu (H.S.); Kenneth.Andries@kysu.edu (K.A.); jerusha.lay@kysu.edu (J.L.); James.adeyemi@kysu.edu (J.A.)

**Keywords:** beef cattle, metabolomics, metagenomics, monensin, rumen fluid

## Abstract

**Simple Summary:**

Monensin can enhance the efficiency of feed utilization by modulating rumen fermentation; however, its effects on rumen function has not been fully described. Thus, this study integrated metagenomics and metabolomics analysis to identify differences in functional attributes and metabolites of rumen microbiota in beef steers fed no or 200 mg/d of monensin. Our results showed differences in relative abundance of functional genes involved in lipid metabolism and amino acid metabolism as well as changes in rumen fluid metabolites and their metabolic pathways. This study revealed a better understanding of the effects of monensin, which may enable more effective use of this additive for beef cattle production.

**Abstract:**

To identify differences in rumen function as a result of feeding monensin to beef cattle, rumen fluid metagenomics and metabolomics analyses were used to evaluate the functional attributes and metabolites of rumen microbiota in beef steers fed no or 200 mg/d of monensin. Eight rumen-fistulated steers were used in the study for a period of 53 days. Rumen fluid samples were collected on the last day of the experiment. Monensin increased the relative abundance of *Selenomonas* sp. ND2010, *Prevotella dentalis*, *Hallella seregens*, *Parabacteroides distasonis*, *Propionispira raffinosivorans*, and *Prevotella brevis*, but reduced the relative abundance of *Robinsoniella* sp. KNHs210, *Butyrivibrio proteoclasticus*, *Clostridium botulinum*, *Clostridium symbiosum*, *Burkholderia* sp. LMG29324, and *Clostridium butyricum*. Monensin increased the relative abundance of functional genes involved in amino acid metabolism and lipid metabolism. A total of 245 metabolites were identified. Thirty-one metabolites were found to be differentially expressed. Pathway analysis of the differentially expressed metabolites revealed upregulated metabolic pathways associated with metabolism of linoleic acid and some amino acids. These findings confirm that monensin affects rumen fermentation of forage-fed beef cattle by modulating the rumen microbiome, and by reducing amino acid degradation and biohydrogenation of linoleic acid in the rumen.

## 1. Introduction

The rumen microbiota plays a central role in the efficiency of digestion in ruminants [1], thus, the use of rumen fermentation modifiers, such as monensin, that can enhance the efficiency of feed utilization by increasing the amount of energy available has been the focus of research for several years [2]. Monensin is a carboxylic polyether ionophore fed to ruminants to modify rumen fermentation dynamics by selectively inhibiting growth of gram-positive bacteria, which produce most of the acetate, lactate, and hydrogen in the rumen [3,4]. This favors growth of gram-negative bacteria and production of propionate in the rumen [5]. Increased production of propionate in the rumen increases hepatic gluconeogenic flux [6], which improves the overall energy status of ruminants [7]. Monensin also decreases amino acid fermentation and ruminal ammonia concentration in the rumen [8], however, despite having a basic understanding of the effects of monensin and the significance of rumen microbiota, little is known about the effects of monensin on the genetic, functional, and metabolomic attributes of the rumen microbiota.

In recent years, the availability of high-throughput comparative metagenomics enabled by development of next-generation sequencing platforms has advanced our understanding of the composition and function of microbial populations in diverse environments [9,10]. Microbial characterization via 16S rRNA has been used to assess the effects of monensin on ruminal bacterial diversity in dairy and beef cattle [4,11]. However, this technique is limited because of the bias induced by PCR while amplifying the gene and it offers limited taxonomical and functional resolution; in general, OTUs are analyzed at the genera level, and can be less precise at the species level [12,13]. Shotgun metagenomic sequencing avoids PCR bias and provides a genetic and functional perspective of the microbiome [14]. This technique has already been used to reveal functional genes and microbiome associated with performance and disease conditions of cattle [15,16]. Application of metabolomics analysis provides an opportunity to measure large numbers of small molecule metabolites in cells, tissues and biofluids comprehensively [17]. Recent studies have applied metabolomics to predict feed efficiency and residual feed intake [18,19], examine disease conditions [20], evaluate dietary responses to different feeds [21], and assess milk quality of ruminants [22].

To date, the influence of dietary monensin on functional and metabolomic attributes of the rumen microbiota has not been studied in beef cattle. Therefore, the objective of this study is to determine the functional analysis of the rumen microbiota and its metabolomic attributes in forage-fed Holstein steers fed no or 200 mg d^−1^ of monensin. In this study, we applied ultra-performance liquid chromatography (UPLC)/time-of-flight mass spectrometry (MS) and multivariate/univariate statistical analysis to characterize rumen metabolites and whole-metagenomic shotgun sequencing to provide a snapshot of the rumen microbial population.

## 2. Materials and Methods

The research protocol (protocol number 18-001) was reviewed and approved by the Institutional Animal Care and Use Committee of Kentucky State University.

### 2.1. Animals, Housing, and Feeding

The trial consisted of a 14-d adaptation to diet and a 39-d treatment period. On day 0, eight rumen-fistulated Holstein steers (593 ± 24 kg body weight) maintained at the Kentucky State University Research Farm were blocked by body weight and assigned randomly to one of two treatments for 39 days: no supplementation (control) and monensin supplementation (200 mg hd^−1^ d^−1^). The steers were housed in individual pens and had *ad libitum* access to water and red clover/orchard grass hay (Table S1). Mineral mix (Hubbard feeds, Mankato, MN, USA) was fed free choice. Concentrate supplement containing corn gluten meal, soy hull, and cracked corn, with no or 200 mg of monensin (Elanco Animal Health, Greenfield, IN, USA) was supplemented at 4 kg/steer daily (Table S1). Steers were fed once daily at 0900 h.

### 2.2. Rumen Fluid Collection

Representative samples (300 mL) of the ruminal contents were collected via the cannula by spot sampling from the midpoint along the length of the ruminal contents and from the midpoint along the height of the ruminal contents (ventral rumen) at approximately 3, 6, and 9 h after feeding on the last day of the experiment. At the time of collection, ruminal contents were tightly hand-strained through 4 layers of sterile cheesecloth to separate the liquid and solid samples. Daily composited samples (solid and liquid samples) were mixed 1:1 (*w*/*w*) and stored at −80 °C for subsequent metagenomics and metabolomics analysis.

### 2.3. Whole-Genome Shotgun Sequencing

#### 2.3.1. DNA Extraction and Sequencing

Frozen rumen fluid samples were thawed at room temperature and centrifuged at 15,000 × *g* followed by removing the supernatant. The DNA was then extracted and purified from the pellets using a PowerSoil DNA Isolation Kit (MO BIO Laboratories Inc., Carlsbad, CA, USA) according to the manufacturer’s instructions. The integrity of the DNA was verified by agarose (0.7%) gel electrophoresis, and the DNA was stored at −20 °C until further use. The sequencing library was constructed by DNA fragmentation following manufacturer’s instructions (Illumina) [23]. Following Library preparation, the sheared DNA fragments were then sequenced on an Illumina HiSeq 2500 platform by an Illumina HiSeq–PE150 bp strategy.

#### 2.3.2. Bioinformatics and Statistical Analysis

After sequencing, the raw sequence data files were demultiplexed and stored as fastq format. The sequence data were assembled using IDBA-UD assembler [24] following quality control such as removal of reads containing barcode and adaptors, low-quality reads, and contaminative reads. MetaGene (http://metagene.cb.k.u-tokyo.ac.jp/) was employed to predict open reading frames from assembled contigs [25], and gene sequence with length more than 100 bp were kept and translated into amino acid sequences. All the predicted genes were then put together and clustered by CD-HIT software (http://www.bioinformatics.org/cd-hit/) with the parameters (95% identity, 90% coverage) [26]. The longest gene was used as a representative sequence to construct a non-redundant gene set. The non-redundant multi-source protein annotation database (M5NR [27]) was used for phylogenetic classification. Based on Kruskal–Wallis (KW) sum-rank test, the linear discriminant analysis effect size (LEfSe) method was used to identify the most differentially abundant taxonomic features at genus and species levels [28]. For this analysis, the significance threshold for the Kruskal–Wallis (KW) test was set to 0.05 and the logarithmic linear discriminant analysis (LDA) score cut-off was set to 2.0. The set of genes was aligned with KEGG (http://www.genome.jp/kegg/) gene database using BLAST to obtain functional annotation information [29]. Gene sequences were aligned with the CAZy database (http://www.cazy.org/) to get information on the functional classification of carbohydrate-active enzymes [30]. Differences in the functional features of the two metagenomes were analyzed using Mann Whitney test using *p*-value ≤ 0.05.

### 2.4. Non-Targeted Metabolomics Analysis

#### 2.4.1. Sample Preparation and Analysis

The rumen fluid samples were thawed on ice at room temperature approximately 2 h before use. The samples were prepared using the procedure of Want et al. [31]. Briefly, 500 µL of the ruminal fluid samples were mixed with 2 mL of methanol-water (1:1, *v*/*v*) and then vortex-mixed for 2 min. Subsequently, the mixture was centrifuged at 15,000× *g* at 4 °C for 10 min. The supernatant was dried in a vacuum concentrator, and re-suspended in 200 µL methanol/water (1:1 vol/vol). The analysis was done using an UltiMateTM 3000 ultra-performance liquid-chromatography (UPLC) system (Thermo Fischer Scientific, Waltham, MA, USA) equipped with an autosampler and coupled with an Orbitrap-Velos mass spectrometer (MS). Chromatographic separation was carried out with an Agilent Extend C-18 column (3.0 × 150 mm, 3.5 µm; Agilent) with a temperature of 45 °C and sample manager temperature of 4 °C. The UPLC mobile phases were (A) 0.1% formic acid-water and (B) 0.1% formic acid-acetonitrile. The injection volume was 5 µL, and the flow rate was 0.5 mL/min. Mass spectrometry was performed in both positive and negative modes. The capillary voltage was 3.5 kV for both positive and negative modes. Capillary and source-heat temperature was set at 350 °C, sheath gas flow at 40 L h^−1^, and auxiliary gas flow at 10 L h^−1^. Quality control (QC) sample was prepared by mixing 10 µL of each rumen fluid sample. The QC was run in positive and negative modes every 4 samples to serve as technical replicates for validating stability and reproducibility of UPLC/MS system.

For analysis of volatile fatty acids (acetate, propionate, and butyrate), 12 μL of 50% H_2_SO_4_ were added to 12 mL of the liquid portion of ruminal content, and the mixture was centrifuged at 11,500× *g* for 20 min. The supernatant was stored at −20 °C until analyzed for volatile fatty acids using a Merck Hitachi Elite La-Chrome High-Performance Liquid Chromatograph system (Hitachi L2400, Tokyo, Japan) fitted with a Bio-Rad Aminex HPX-87H column (Bio-Rad Laboratories, Hercules, CA, USA) with a 0.015 M sulfuric acid mobile phase and a flow rate of 0.7 mL/min at 50 °C [32].

#### 2.4.2. Data Processing and Statistical Analysis

The raw data were converted to Analysis Base File format by Reifycs ABF Converter (http://www.reifycs.com/AbfConverter/index.html). Processing of the raw data, including peak picking, deconvolution, compound identification, and peak alignment was done using the MS-DIAL version 2.84 [33] based on the mass to charge (*m*/*z*) value and the retention time of the ion signals. Identified metabolites from both positive and negative modes were merged and imported into the MetaboAnalyst 4.0 [34] for multivariate/univariate analysis. After normalization, Principal Components Analysis (PCA) was first used as an unsupervised method for data visualization and outlier identification. Supervised regression modeling was then performed by the use of orthogonal partial least squares discriminant analysis (OPLS-DA) to identify the significantly differential metabolites. The differential metabolites were filtered and confirmed by combining the results of t-test (*p* ≤ 0.10) and fold change (FC) of the peak intensities (mean value of peak intensity obtained from monensin group/mean value of peak intensity obtained from Control group). The chemical structures of differential metabolites were identified according to online databases such as the Human Metabolome Database (www.hmdb.ca), Metlin (www.metlin.scripps.edu) and the Mass Bank (www.massbank.jp) using the data of accurate masses and MS/MS fragments. Pathway analysis was conducted using MetaboAnalyst 4.0 software with a *Bos taurus* pathway library using hypergeometric test for over-representation analysis and relative-betweeness centrality for pathway topology analysis.

## 3. Results

### 3.1. Rumen Fluid Metagenomics Profiling

#### 3.1.1. Sequencing Results

Metagenome sequencing of rumen fluid samples from the 8 Holstein beef steers yielded approximately 24.1 million reads per sample, which resulted in about 84,033 contigs per sample. Rarefaction analysis, which was used to assess the depth of sequencing, showed that the number of sequences used for all samples was sufficient to determine the total number of sequence types (Figure S1).

#### 3.1.2. Phylogenetic Profile

**Figure 1:**
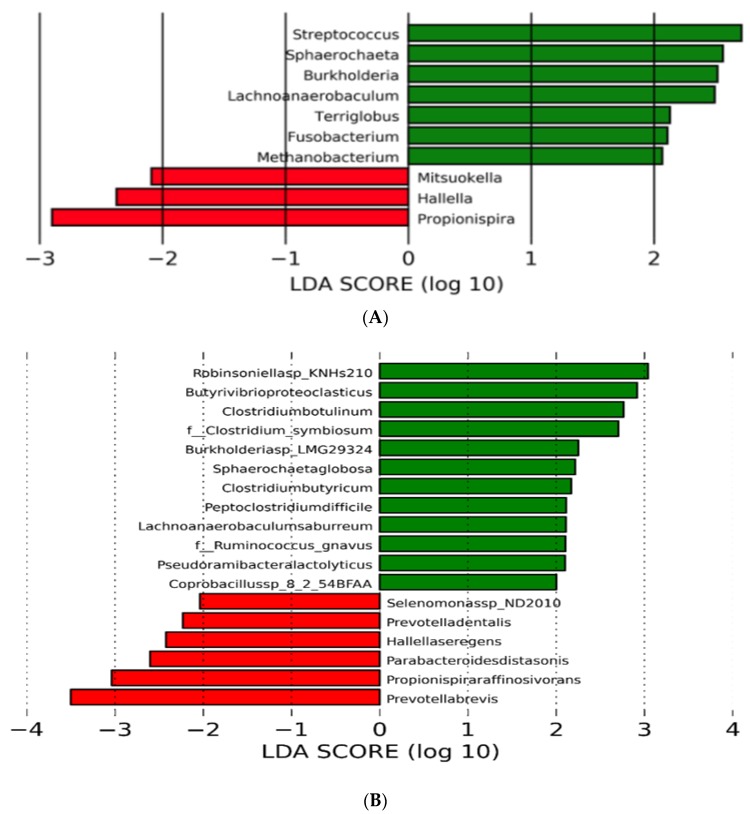
Linear discriminant analysis effect size (LEfSe) of rumen microbiota of beef steer fed no (control) or 200 mg/d of monensin. The linear discriminant analysis plot indicates the most differentially abundant taxa found by ranking according to their effect size (≥2.0) at the genus (**A**) and species level (**B**). The taxa enriched in steers fed the control diet are indicated with a positive score (green), and taxa enriched by the monensin treatment have a negative score (red). Only taxa meeting the significant threshold of 2.0 are shown.

Sequences from the rumen fluid samples predominantly contained phylotypes affiliated with *Bacteroidetes* (39.4 ± 5.3%), *Firmicutes* (15.9 ± 2.0%), *Proteobacteria* (1.9 ± 0.9%), *Actinobacteria* (0.6 ± 0.08%), and *Euryarchaeota* (1.0 ± 0.9%) (Table S2). Though no statistical difference was found, the relative abundance of *Bacteroidetes* was greater in steers fed monensin (43.5 ± 3.5% vs. 35.3 ± 3.1%) (Table S2). At the genus level, 1541 taxa were detected. *Prevotella* (24.7 ± 5.05%) was the most predominant, followed by *Bacteroides* (6.63 ± 1.38%) (Table S3). At the species level, 7105 taxa were detected (Table S4). The most predominant were *Prevotella* sp. FD3004 (6.41 ± 1.96%), followed by *Prevotella ruminicola* (2.93 ± 0.58%), *Prevotella* sp. MA2016 (1.86 ± 0.47%), and *Prevotella brevis* (1.70 ± 0.30%) (Table S4). To identify differentially abundant taxa that were mostly affected by treatment with monensin, we compared the rumen microbial population of both treatments using a metagenomic biomarker discovery approach, LEfSe, which performs a nonparametric Wilcoxon sum-rank test followed by linear discriminant analysis to assess the effect size of each differentially abundant taxon [34]. Using LEfSe, no treatment effects were found at the phylum level. At the genus level, the relative abundance of *Mitsuokella*, *Hallella*, and *Propionispira* was enriched in steers fed monensin, while *Streptococcus*, *Sphaerochaeta*, *Burkholeria*, *Lachnoanaerobaculum*, *Terriglobus*, *Fusobacterium*, and *Methanobacterium* were reduced (Figure 1A). At the species level, the relative abundance of 616 taxa were affected by treatment (data not shown). However, LEfSe analysis of the 500 most predominant species revealed that the relative abundance of *Selenomonas* spp. ND2010, *Prevotella dentalis*, *Hallella seregens*, *Parabacteroides distasonis*, *Propionispira raffinosivorans*, and *Prevotella brevis* were enriched in steers fed monensin, while the relative abundance of 12 taxa, such as *Robinsoniella* sp. KNHs210, *Butyrivibrio proteoclasticus*, *Clostridium botulinum*, *Clostridium symbiosum*, *Burkholderia* sp. LMG29324, and *Clostridium butyricum*, reduced (Figure 1B).

#### 3.1.3. Functional Profile of the Ruminal Microbial Community

**Figure 2:**
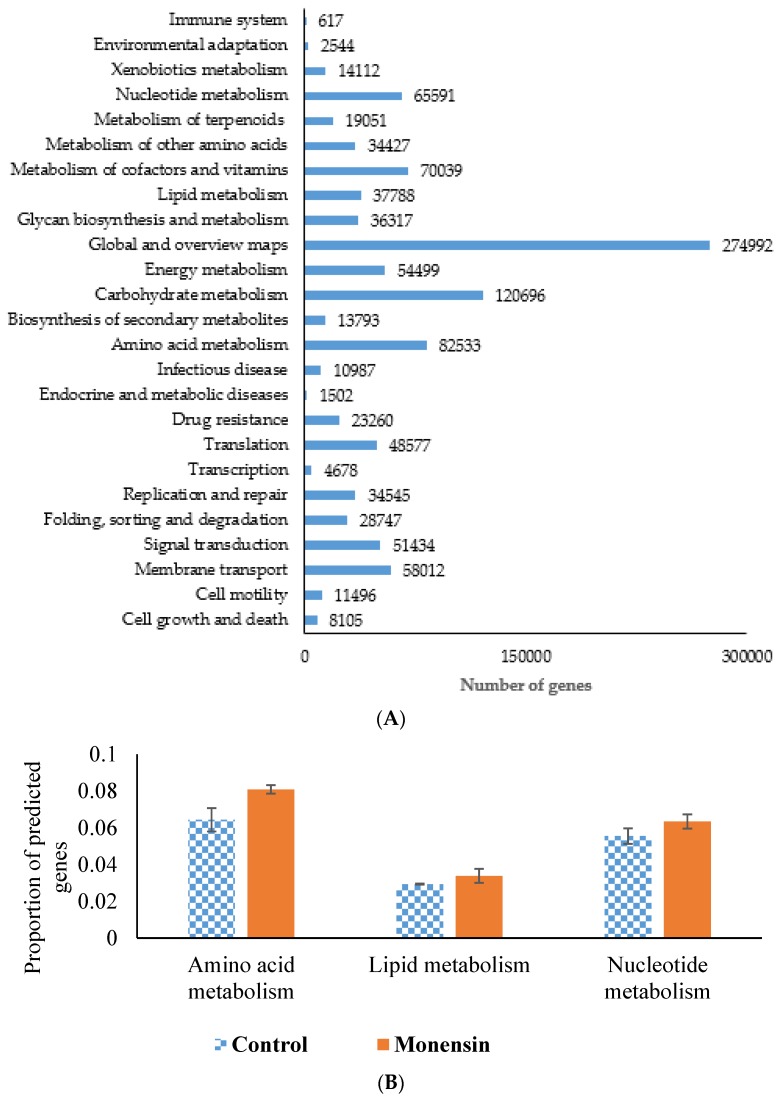
(**A**) Distribution of category of the predicted genes by KEGG. (**B**) Differential KEGG gene functions. Differences between control and monensin samples were tested for significance using a Mann Whitney test (*p* ≤ 0.05).

Using KEGG orthology level 2, the most predominant genes were those involved in metabolism, including amino acid metabolism, carbohydrate metabolism, glycan biosynthesis and metabolism, lipid metabolism, metabolism of cofactors and vitamins, xenobiotics biodegradation and metabolism, and nucleotide metabolism (Figure 2A). Other predominant genes were those involved in membrane transport, signal transduction, replication and repair, translation, and folding, sorting, and degradation (Figure 2A). The relative abundance of functional genes involved in amino acid metabolism, nucleotide metabolism, and lipid metabolism were enriched (*p* < 0.05) in the metagenome of steers fed dietary monensin (Figure 2B).

**Figure 3:**
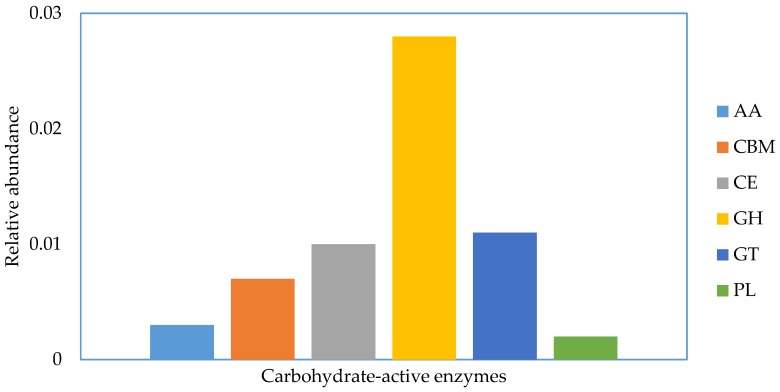
Relative abundance of the category of carbohydrate-active enzymes according to carbohydrate-active enzyme (CAZy) database. AA = Auxilliary Activities, CBM = Carbohydrate Binding Modules, CE = Carbohydrate Esterases, GH = Glycoside Hydrolases, GT = Glycosyl Transferases, PL = Polysaccharide Lyases.

The genes aligned to the CAZy database were categorized into 6 types, auxiliary activities, carbohydrate-binding modules, carbohydrate esterases, glycoside hydrolases, glycoside transferases, and polysaccharide lyases. Carbohydrate hydrolases were the most enriched, followed by glycosyl transferases and carbohydrate esterases (Figure 3). No effects of dietary treatment on the relative abundance of carbohydrate-active enzymes were detected.

### 3.2. Rumen Fluid Metabolomics Profiling

Quality control samples were used to demonstrate the stability of the UPLC/MS system. The clustering of QC samples in the PCA scores plot (Figure S2) demonstrated satisfactory reproducibility and stability of the metabolic features and were subsequently used for statistical analysis.

**Figure 4:**
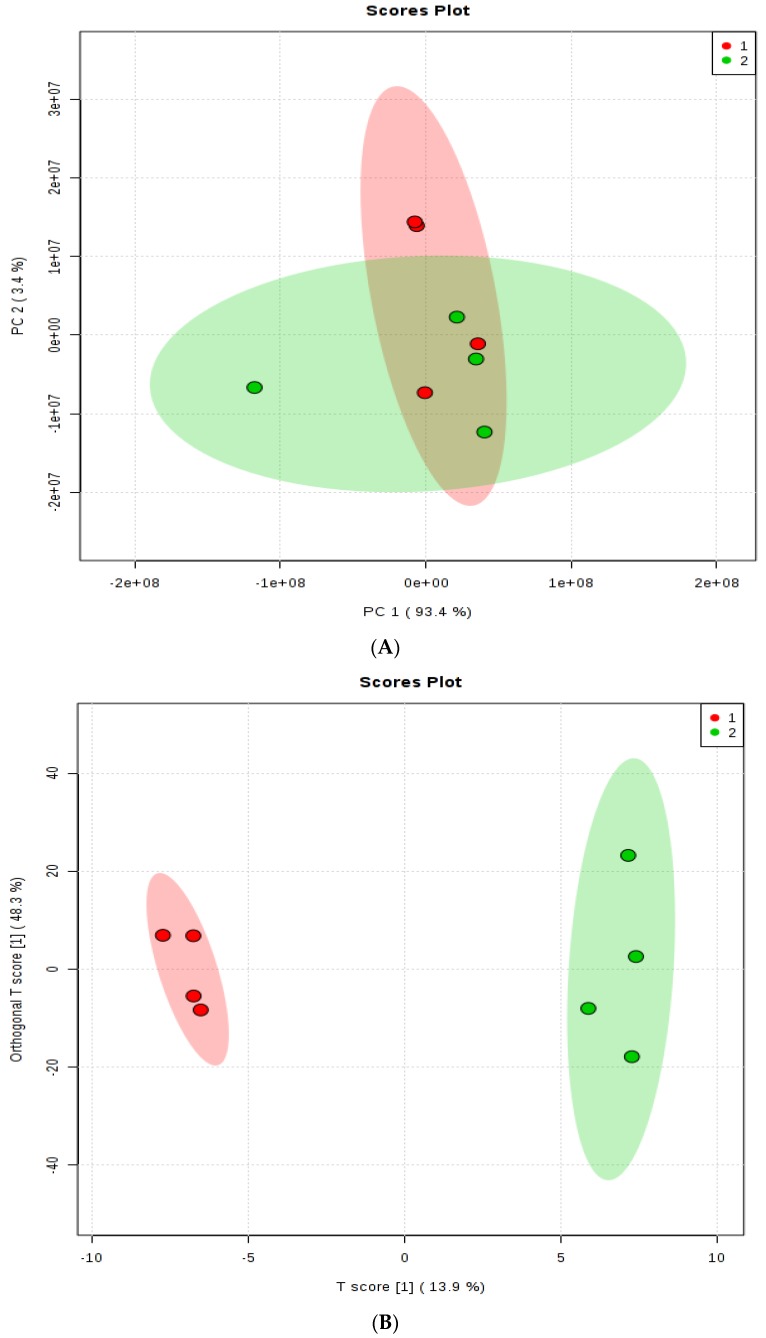
(**A**) The scores plot of PCA model showing the directions that best explain the variance between the two treatments. (**B**) OPLS-DA score plot of all metabolite features. Group 1 = steers fed Control diet, Group 2 = steers fed 200 mg d^−1^ of monensin. One data point represents one composite rumen fluid sample of each steer.

In total, 245 metabolites were identified (Table S5). Principal component analysis revealed different distributions of the two treatments. PC1 explained 93.4% and PC2 explained 3.4% of the variance (Figure 4A). Score plot from OPLS-DA, a powerful tool used for dimension reduction and identification of spectral features that drive group separation [35], revealed a clear separation of the two treatments (Figure 4B), indicating that feeding dietary monensin altered the ruminal metabolome. Permutation analysis (*p*-value = 0.05, R^2^ = 0.99, Q^2^ = 0.42) of the OPLS-DA confirmed the validity of the model.

Thirty-one metabolites were differentially expressed (*p* ≤ 0.10; Table 1). Feeding dietary monensin increased (*p* ≤ 0.10; FC > 1.0) the relative concentrations of 24 metabolites, such as linoleic acid, l-phenylalanine, hypoxanthine, oxysporidinone, l-β-homomethionine, 2-methylgluric acid, isopongaflavone, catechol, pimelic acid, l-Histidine, tri-*O*-methylgenistein, oxysporidinone, formylindole, indole-3-carboxylic acid, and 8,15-DiHETE. The relative concentrations of acetate, 4-aminophenol, tyramine, 3-(4,4-dimethyl-4,5-dihydro-1,3-oxazol-2-yl)pyridine, 4-pyrodoxic acid, 1,3-dipropyl-7-methylxanthine, pentalenolactone, and vildagliptin, and apigenin were reduced (*p* ≤ 0.10; FC < 1.0) in steers fed monensin. Results of volatile fatty acid profile revealed that monensin reduced acetate (52.3 vs. 60 mM; *p* ≤ 0.05), but increased propionate (27.5 vs. 23.3 mM) compared with the control.

Pathway analysis of the differentially expressed metabolites revealed 14 associated metabolic pathways. Metabolic pathways associated with linoleic acid metabolism (impact value = 1.0), phenylalanine, tyrosine and tryptophan biosynthesis (impact value = 0.5), phenylalanine metabolism (impact value = 0.41), and histidine metabolism (impact value = 0.27) were upregulated in steers fed monensin (Table 2).

## 4. Discussion

The rumen microbiota plays a central role in the efficiency of digestion in ruminants [36]. Thus, determining the functional and metabolomic attributes of the rumen microbiome is essential for understanding their role on host metabolism and health [36,37]. Manipulation of the rumen microbiota via dietary intervention, such as the use of monensin with the aim of improving the host performance has been an active area of research for several years [38]. In this study, we used shotgun metagenomics sequencing and KEGG gene annotation database to evaluate the functional potential of the rumen microbiota, as well as UPLC/MS method and pattern recognition approaches, such as PCA and OPLS-DA to identify differences in rumen metabolites in beef cattle fed no or 200 mg d^−1^ of monensin. To the best of our knowledge, this is the first report evaluating the effects of dietary monensin on the functional and metabolomic attributes of the rumen microbiota in forage-fed beef cattle.

It is notable that the rumen microbial composition and the relative abundance of phyla is influenced by diet [16,39]. Similar to other studies [39,40], the predominant phyla identified in this study include *Bacteroidetes*, *Firmicutes*, *Proteobacteria*, and *Euryarchaeota*. The numerically higher relative abundance of gram-negative *Bacteroidetes* and lower relative abundance of gram-positive *Firmicutes* in steers fed monensin in this study is in accordance with the mode of action of monensin, which acts by reducing the population of gram-positive bacteria in the rumen. A recent study that characterized the gut microbiome of feedlot cattle supplemented with an antibiotic feed additive containing tylosin and monensin had similar results [16]. In addition, a study that investigated the ruminal bacterial diversity in dairy cattle fed monensin indicated the proportion of *Firmicutes* was reduced while that of *Bacteroidetes* was increased [11].

We used the LEfSe method to identify those taxa that are affected by the dietary treatment at the genus and species levels. LEfSe combines statistical significance, biological relevance, and effect size using linear discriminant analysis to identify genomic features such as genes, pathways, or taxa characterizing the differences between two or more biological conditions [28]. In this study, both gram-positive and gram-negative taxa were reduced in response to monensin. This agrees with the suggestion that the presence or absence of an outer membrane is not the only factor that determines susceptibility of a bacterium to monensin [11]. Future studies should evaluate other factors that may alter the effect of monensin on rumen bacteria such as chemicophysical features of solid feed particles and the ability to adapt to ionophores [11].

It should be noted that all of the taxa enriched in response to monensin treatment are gram-negative, confirming that the overall effects of monensin favor the growth and activities of gram-negative bacteria. *Prevotella* sp., *Hallella seregens*, and *Parabacteroides distasonis* produce succinate [41,42,43], *Selenomonas* species have the ability to decarboxylate succinate to form propionate [44], and *Propionispira raffinosivorans* is a major propionic acid-producer [45]. The shift in the microbial population in favor of the aforementioned bacteria supports the increased propionate synthesis observed in this study as a result of feeding monensin. Similar to the results of this study, several studies have reported the effectiveness of dietary monensin at altering the production and proportion of rumen fermentation acids. A recent study reported increased propionate concentrations in forage-fed beef steers consuming 200 mg d^−1^ of monensin [46]. Similarly, beef cattle consuming low-quality dry winter range grass fed 200 mg d^−1^ of monensin had a decrease in the molar percentage of acetate and an increase in the molar percentage of propionate in [47]. The relative proportion of fermentation acids produced in the rumen is significant due to the role of propionate in energy metabolism [7]. In ruminants, propionate is the most predominant substrate for gluconeogenesis, a major pathway for maintaining adequate glucose supply for ruminants [48]. Therefore, increased propionate production in the rumen as a result of feeding monensin is beneficial for cattle.

Another aspect of monensin inclusion worthy of attention is its effect on ruminal methane production. It is hypothesized that monensin can reduce methane production by inhibiting the growth of the bacteria responsible for supplying methanogens with the primary substrates, H_2_ and formate, for methanogenesis [49]. The reduced abundance of *Methanobacterium* by monensin in this study may be as a result of reduced availability of H_2_ and/or differences in growth kinetics of methanogen species because ionophores are not known to inhibit methanogens directly, but can change the population dynamics of methanogen species [50].

In a comprehensive study of the bovine ruminal fluid metabolome, Saleem et al. [21] combined nuclear magnetic resonance spectroscopy, inductively coupled plasma mass-spectroscopy, gas chromatography-mass spectrometry, direct flow injection mass spectrometry and lipidomics with computer-aided literature mining to identify and quantify about 246 ruminal fluid metabolites. In accordance with this study, the composition of bovine ruminal fluid is dominated primarily by microbial metabolites, including short-chain fatty acids, phospholipids, dicarboxylic acids, amino acids, and triglycerides [21]. The alteration in the rumen metabolome we observed as a result of feeding monensin was not surprising since alteration of the rumen microbial population generally influences the types of compounds produced by the rumen microbiota. In this study, the relative concentrations of 31 of the total 247 metabolites identified were altered by feeding monensin.

Pathway analysis of the metabolites expressed differentially in monensin-treated cattle, based on t-test and FC analysis, revealed fourteen pathways were affected: purine metabolism, tyrosine metabolism, vitamin B6 metabolism, phenylalanine, tyrosine and tryptophan biosynthesis, phenylalanine metabolism, nitrogen metabolism, linoleic acid metabolism, biosynthesis of unsaturated fatty acids, aminoacyl-tRNA biosynthesis, histidine metabolism, glycolysis or gluconeogenesis, propanoate metabolism, pyruvate metabolism, and caffeine metabolism. However, only four pathways (phenylalanine, tyrosine and tryptophan biosynthesis, phenylalanine metabolism, histidine metabolism, and linoleic acid metabolism) had pathway impact values greater than 0.1, which represents the cut-off for relevance [51]. All of the relevant pathways were upregulated in steers fed monensin due to increased concentrations of the associated metabolites: phenylalanine, histidine, and linoleic acid. This is in agreement with the KEGG functional gene annotation of the metagenome, which revealed higher relative abundance of functional genes involved in amino acid metabolism and lipid metabolism in steers fed monensin.

The increased abundance of functional genes involved in lipid metabolism and the upregulation of the linoleic acid metabolism pathway in steers fed monensin confirm results from previous studies that suggest monensin can inhibit ruminal biohydrogenation of unsaturated fatty acids [52]. As shown in the current study, several lines of evidence indicate monensin can inhibit the growth of *Butyrivibrio* sp., a group of gram-positive bacteria that can biohydrogenate linoleic acid in the rumen [44,53]. Relative abundance of *Butyrivibrio proteoclasticus*, which is known to biohydrogenate conjugated linoleic acid (CLA) and vaccenic acid in the rumen [54], was reduced in the current study. Fellner et al. [52] observed higher concentrations of vaccenic acid and CLA in continuous culture of bacteria after treatment with monensin. In Korean native steers fed concentrated feed supplemented with monensin, concentrations of cis-9, trans-11 CLA were increased [55]. Consequently, ruminants receiving dietary monensin have higher concentrations of CLA in adipose tissue and milk [56,57]. Odongo et al. [58] and Silva-Kazama et al. [59] reported increased concentration of CLA in milk of dairy cows fed monensin. Likewise, the use of 230 mg/d of monensin increased the concentration of α-linolenic acids in subcutaneous fat of young bulls [60]. Due to the numerous health benefits of CLA for humans, nutritional strategies that increase the CLA content of animal products are considered desirable [58,61].

As shown in this study, monensin can reduce amino acid degradation and ammonia accumulation in the rumen [62,63] due to suppression of hyper-ammonia-producing bacteria, *Peptostreptococcus* and *Clostridium*, in the rumen [64,65,66]. A significant reduction of amino acid degradation was observed in mixed and pure cultures of rumen bacteria treated with monensin [67]. In another study, monensin decreased ammonia concentrations and increased the flow of amino acids from the rumen in cows fed timothy hay supplemented with casein, gelatin, or soy hydrolysate [66]. The reduced abundance of amino acid fermenting bacteria as well as upregulation of phenylalanine, tyrosine, and tryptophan biosynthesis, phenylalanine, and histidine metabolism pathways observed in this study suggests that monensin can reduce wasteful degradation of amino acids in the rumen.

Metagenomics approach requires sophisticated computational methods which are reliant on the integrity and presence of sequence data within established databases that may be subject to low taxonomic resolution [68,69]. Non-targeted metabolomics approach relies on comparing peak intensity to evaluate differences in relative abundance of metabolites which may often lack accuracy and precision [70]. Also, it is still a challenge to accurately identify metabolites due to the complexity and chemical diversity of the metabolome [71]. Another limitation of this study is the limited number of animals used due to high cost of shotgun metagenomic sequencing and LC-MS-based metabolomics. Despite these limitations, this study enhances our understanding of the effects of monensin and confirms the usefulness of metagenomics and metabolomics analyses in ruminant nutrition studies.

## 5. Conclusions

This study showed that feeding monensin altered functional and metabolomic attributes of the rumen microbiota in forage-fed beef cattle. Differences in relative abundance of functional genes involved in lipid metabolism and amino acid metabolism as well as changes in rumen fluid metabolites and their pathways revealed that dietary monensin affects rumen fermentation of forage-fed beef cattle by altering the rumen volatile fatty acid profiles in favor of propionate production, by reducing amino acid degradation, and by manipulating biohydrogenation of unsaturated fatty acid in the rumen.

## Figures and Tables

**Table 1:** Fold changes of differential rumen fluid metabolites in beef steers fed no or 200 mg d^−1^ of monensin.

Metabolite	FC	*p*-Value
Acetate (mM)	0.87	0.05
4-aminophenol	0.80	0.09
Trans-cinnamaldehyde	1.31	0.07
Hypoxanthine	1.20	0.08
Tyramine	0.90	0.07
Formylindole	1.37	0.09
Indole-3-carboxylic acid	1.44	0.05
l-β-homomethionine	1.75	0.03
3-(4,4-dimethyl-4,5-dihydro-1,3-oxazol-2-yl)pyridine	0.81	0.05
4-pyridoxic acid	0.80	0.07
Simazine	1.23	0.03
1,3-dipropyl-7-methylxanthine	0.65	0.02
2-(1,3-benzodioxol-5-yl)ethyl-4-methoxy-2H-pyranone	1.45	0.09
Pentalenolactone	0.65	0.02
l-histidine	1.76	0.04
Vildagliptin	0.70	0.06
Oxybuprocaine	1.41	0.05
Tri-*O*-methylgenistein	1.48	0.01
Isopongaflavone	1.56	0.07
Daphnoretine acetate	1.48	0.02
l-phenylalanine	1.96	0.01
Oxysporidinone	1.24	0.02
Catechol	1.42	0.03
2-methylgluric acid	1.13	0.08
Oxypurinol	1.23	0.02
protocatechuic acid	1.39	0.01
Pimelic acid	1.38	0.01
Isobutylglutaric acid	1.13	0.05
Apigenin	0.80	0.06
Linoleic acid	1.61	0.03
Eriodictyol 7,3′-dimethyl ether	1.17	0.09
8,15-DiHETE	1.48	0.02

FC = fold change, mean value of peak intensity obtained from monensin group ÷ mean value of peak intensity obtained from control group. FC values >1 means that metabolite is greater in steers fed monensin and FC values < 1 means that metabolite is lower in steers fed monensin.

**Table 2:** Metabolic pathway analysis of the differential metabolites.

Annotation	Impact	*p*-Value	TC	Hit
Caffeine metabolism	0	0.15	12	1
Purine metabolism	0.01	0.62	68	1
Tyrosine metabolism	0.03	0.44	42	1
Aminoacyl-tRNA biosynthesis	0	0.22	64	2
Vitamin B6 metabolism	0	0.12	9	1
Phenylalanine, tyrosine and tryptophan biosynthesis	0.5	0.05	4	1
Phenylalanine metabolism	0.41	0.12	9	1
Linoleic acid metabolism	1.00	0.06	5	1
Biosynthesis of unsaturated fatty acids	0	0.41	42	1
Histidine metabolism	0.27	0.17	14	1
Nitrogen metabolism	0	0.11	9	1
Glycolysis or gluconeogenesis	0.03	0.30	26	1
Pyruvate metabolism	0.06	0.26	22	1
Propanoate metabolism	0	0.24	20	1

Impact = pathway impact value calculated from pathway topology analysis; TC = total number of compounds in the pathway; Hit = matched number from the uploaded metabolite data.

## Supplementary Materials

The following are available online at http://www.mdpi.com/2076-2615/8/11/211/s1, Table S1: Nutritional composition of the diet, Table S2: Average relative abundance of taxa at the genus level, Table S3: Average relative abundance of taxa at the genus level, Table S4: Average relative abundance of taxa at the genus level, Table S5: List of identified metabolites, Figure S1: Rarefaction curve of the 8 samples, Figure S2. The scores scatter plot of the PCA model showing the clustering of the quality control samples.
