# Supplementary material for: Monensin Alters the Functional and Metabolomic Profile of Rumen Microbiota in Beef Cattle
Source: Animals (Basel). 2018 Nov 17;8(11):211. doi: 10.3390/ani8110211 (PMC6262558; doi:10.3390/ani8110211)
Supplement: Supplementary file 1 [file animals-08-00211-s001.zip › Supplementary FigureS2.pdf]

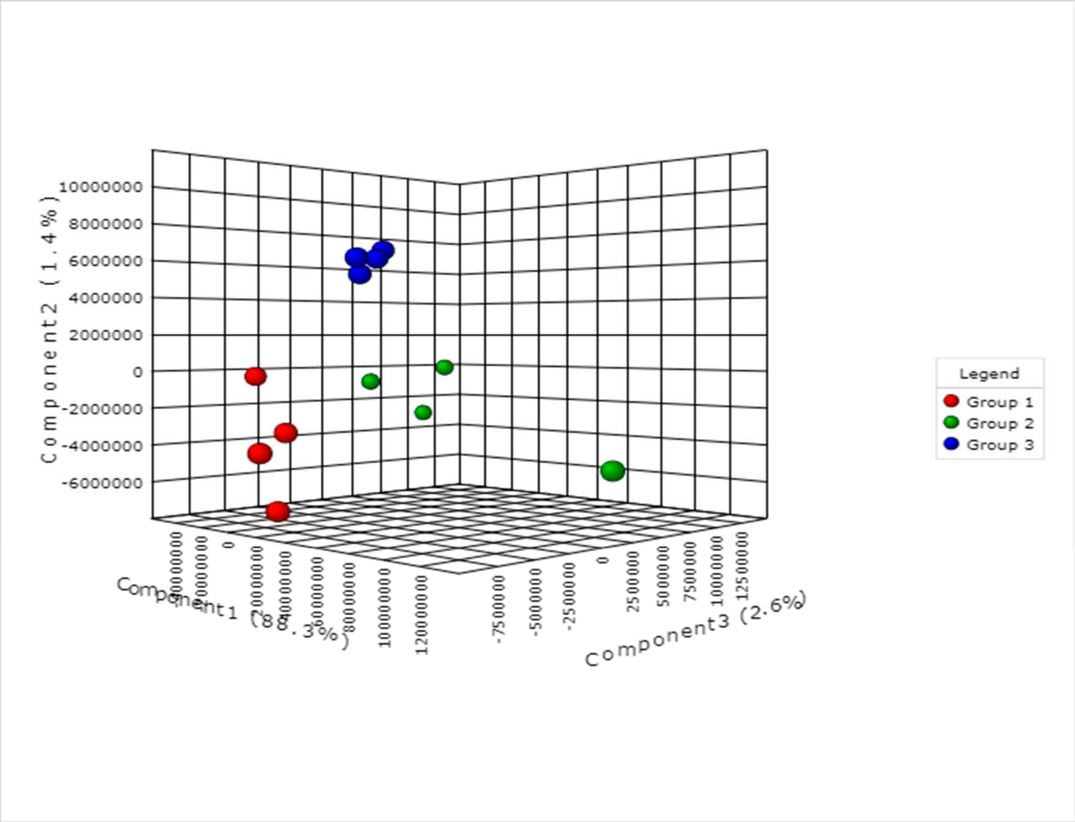

Figure S2. The scores scatter plot of the PCA model showing clustering of the quality control samples; Group 1 = Control samples, Group 2 = Monensin samples, Group 3 = Quality control samples.
