# Supplementary material for: Monensin Alters the Functional and Metabolomic Profile of Rumen Microbiota in Beef Cattle
Source: Animals (Basel). 2018 Nov 17;8(11):211. doi: 10.3390/ani8110211 (PMC6262558; doi:10.3390/ani8110211)
Supplement: Supplementary file 1 [file animals-08-00211-s001.zip › Supplementary Table S1.pdf]

Table S1. Nutritional composition of the diet

	Red clover/orchard grass hay mixture	Concentrate supplement
Dry matter (%)	92.6	89.3
NDF (% DM)	58.9	45.3
ADF (% DM)	40.2	24.4
Crude protein (% DM)	11.4	14.3
Ether extract (% DM)	NA[1]	2.44
Starch (% DM)	NA	23.6

NDF; neutral detergent fiber, ADF; acid detergent fiber

Concentrate contains corn gluten meal, soyhull, and cracked corn in equal proportions.

Guaranteed analysis of the mineral mix (Hubbard feeds, Mankato, MN); 8.0% calcium, 6% phosphorus, 14% magnesium, 12 ppm cobalt, 2000 ppm copper, 55 ppm iodine, 4800 ppm manganese, 36.4 ppm selenium, 4800 ppm zinc, 100,000 IU/lb vitamin A, 20,000 IU/lb vitamin D, and 250 IU/lb vitamin E.

[1]Not measured
