# Supplementary figures and images for: Monensin Alters the Functional and Metabolomic Profile of Rumen Microbiota in Beef Cattle
Source: Animals (Basel). 2018 Nov 17;8(11):211. doi: 10.3390/ani8110211 (PMC6262558; doi:10.3390/ani8110211)

Figure S1. Rarefaction curve of the 8 samples

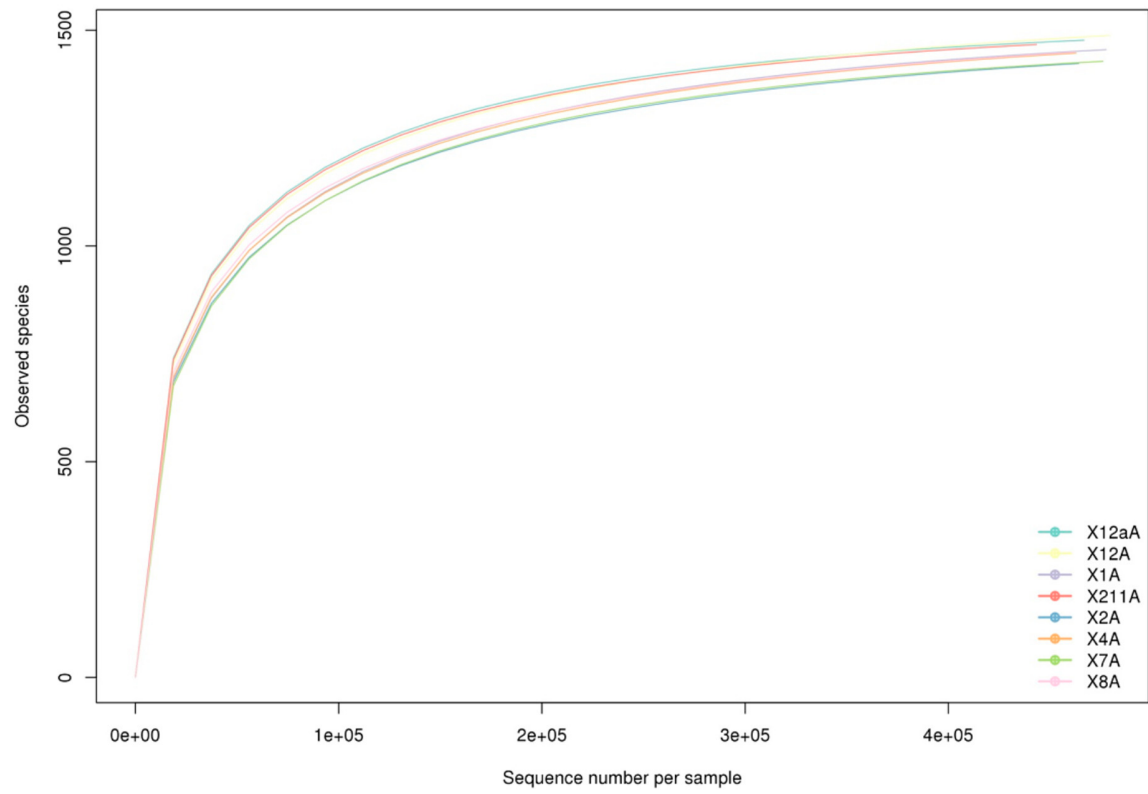

Supplement: Supplementary file 1 [file animals-08-00211-s001.zip › Supplementary FigureS1.pdf]
